# Supplementary material for: Targeting Host PIM Protein Kinases Reduces Mayaro Virus Replication
Source: Viruses. 2022 Feb 18;14(2):422. doi: 10.3390/v14020422 (PMC8878588; doi:10.3390/v14020422)
Supplement: Supplementary file 1 [file viruses-14-00422-s001.zip › viruses-1569753-supplementary.pdf]

viruses

MDPI

Supplementary Materials

Targeting Host PIM Protein Kinases Reduces Mayaro Virus Replication

Madelaine Sugasti-Salazar [1,2], Dalkiria Campos [1], Patricia Valdés-Torres [1], Paola Elaine Galán-Jurado [1] and José González-Santamaría [1,*]

[1] Grupo de Biología Celular y Molecular de Arbovirus, Instituto Conmemorativo Gorgas de Estudios de la Salud, Panamá 0816-02593, Panamá,; msugasti@gorgas.gob.pa (M.S.-S.); dcampos@gorgas.gob.pa (D.C.); pavt2099@gmail.com (P.A.V.-T.); paola.elaine.jurado@gmail.com (P.E.G.-J.)

[2] Programa de Maestría en Microbiología Ambiental, Universidad de Panamá, Panamá 3366, Panamá

* Correspondence: jgonzalezsantamaria@gorgas.gob.pa; Tel.: +507 527-4814

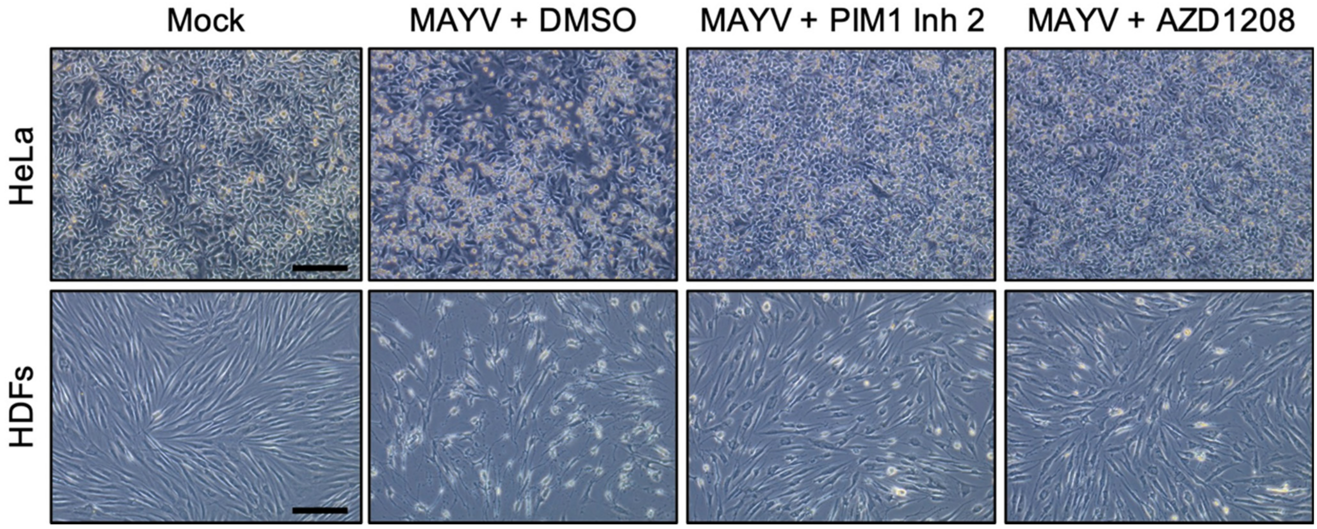

Figure S1. PIM kinase inhibitors partially prevent MAYV-induced cytopathic effects. HeLa cells or HDFs were pre-treated with PIM1 Inh 2, AZD1208 or DMSO (as a control) for 2 h. Then, the compounds were removed and the cells were infected with MAYV at an MOI of 1 (HDFs) or 10 (HeLa cells). After 1 h of virus absorption, virus was removed and PIM kinase inhibitors or DMSO were added to the cells in fresh medium and incubated for an additional 48 h. Next, cytopathic effects were evaluated with an inverted microscope and an MCI70-HD camera (Leica). Scale bar: 100 μm.

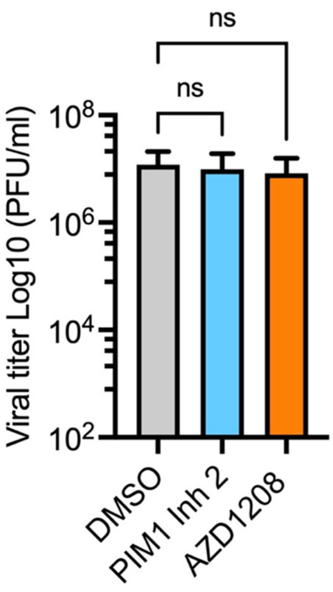

Figure S2. PIM kinase inhibitors have no virucidal effect on MAYV. 1 × 10^7 PFU of MAYV were incubated with PIM kinase inhibitors in serum-free medium for 2 h at 37 °C. Next, the virus was directly titrated using a plaque-forming assay. Data were analyzed with the Mann & Whitney test. Statistically significant differences are denoted as follows: ns: non-significant.
